# How Does Allergen Sensitization Affect Orthodontic Tooth Movement-Associated Phenomena? A Systematic Review of Animal Studies

**DOI:** 10.3390/dj13040166

**Published:** 2025-04-15

**Authors:** Fatima Saeed Bineshaq, Athanasios E. Athanasiou, Miltiadis A. Makrygiannakis, Sotirios Kalfas, Eleftherios G. Kaklamanos

**Affiliations:** 1 Hamdan Bin Mohammed College of Dental Medicine, Mohammed Bin Rashid University of Medicine and Health Sciences, Dubai P.O. Box 505055, United Arab Emirates; fatima.bineshaq@alumni.mbru.ac.ae (F.S.B.);; 2 School of Dentistry, European University Cyprus, 2404 Nicosia, Cyprus; 3 School of Dentistry, National and Kapodistrian University of Athens, 11527 Athens, Greece; 4 School of Dentistry, Aristotle University of Thessaloniki, 54124 Thessaloniki, Greece

**Keywords:** allergy, allergen sensitization, orthodontics, dentistry, systematic review

## Abstract

**Background:** The immune reactions of patients suffering from chronic allergies and asthma are associated with systemic imbalances that may lead to the overexpression of mediators potentially involved in bone remodeling during orthodontic tooth movement. **Aim:** The aim of this systematic review was to evaluate the existing evidence from animal studies with regard to the effects of allergen sensitization on the phenomena correlated with orthodontically induced tooth movement. **Materials and Methods:** This systematic review was based on PRISMA 2020 guidelines. A search without restrictions and hand searching were performed from inception to December 2024. The investigation focused on the impact of allergen sensitization on phenomena associated with orthodontic tooth movement. After the retrieval and selection of relevant studies, data extraction was performed, and the data’s risk of bias was evaluated with the SYRCLE’s Risk of Bias Tool. **Results:** From the detected records, the inclusion criteria were met by only three studies. At the beginning of tooth movement, periodontal ligament was found to be more compressed in the stress area and more stretched in the tension area in sensitized animals. The amount of tooth movement after 14 days of force application was also greater. However, there were conflicting outcomes regarding root resorption. The risk of bias in the retrieved studies was assessed as high overall. **Conclusions:** Despite the fact that existing evidence is not directly related to human beings and is based on a limited number of animal studies, allergen sensitization could potentially influence the phenomena associated with orthodontic tooth movement, and orthodontists should be aware of the relevant implications.

## 1. Introduction

Orthodontic tooth movement (OTM) is initiated by mechanical strains that move a tooth within its surrounding periodontal ligament (PDL) space and form areas of periodontal fiber compression and stretching [1]. These mechanical perturbations, combined with variations in blood flow and oxygen availability, result in the aseptic activation of complex signaling pathways [1]. Eventually, mechanical stimuli lead to the release of molecular mediators, including factors implicated in the processes of inflammation, which induce the differentiation of cells into osteoclasts and osteoblasts. These cells orchestrate a highly harmonized combination of alveolar bone resorption and deposition and, ultimately, the movement of teeth [1].

The abnormal stimulation of odontoclastic cells during OTM might lead to orthodontically induced root resorption (OIRR) [2]. Odontoclasts present significant morphologic and functional similarities with osteoclasts but are specialized in the degradation of the mineral and organic components of the dentine [3,4]. Moderate generalized OIRR is common but without clinical significance [5]. Sporadically, the usually self-regulated odontoclastic activation [2] results in significant reductions in the root length of certain teeth and a greater crown-to-root ratio with clinical repercussions [6,7]. Various parameters have been considered to predispose patients to OIRR. Some of them are related to orthodontic treatment per se, whereas others are patient-related, like genetic background, gender, age, root morphology, history of trauma, and systemic factors [8,9]. The systemic factors that have been reported to increase one’s susceptibility to OIRR include certain medications, hormonal imbalances, and metabolic derangements, like asthma and allergic diseases [10,11,12,13].

Asthma and allergic conditions represent significant global public health issues with socio-economic implications and a notable rise in incidence over recent decades [14,15]. The immune responses of patients with such conditions are marked by metabolic imbalances, which lead to an increased production of inflammatory factors [16,17]. These molecules can potentially enter the bloodstream, reach sites where bone is remodeled under the influence of orthodontic forces, and affect the differentiation and activation of clastic cells and ultimately the development of OIRR [12,13]. Evidence regarding the associations of a medical history of asthma or allergy with the prevalence of OIRR in humans remains inconclusive [18,19].

The present systematic review on animal studies focuses on the relationship between allergen sensitization and the phenomena associated with orthodontic tooth movement, with a particular emphasis on orthodontically induced root resorption.

## 2. Materials and Methods

### 2.1. Protocol Development

Relevant methodological guidelines were followed to develop, conduct, and report on the protocol and the report of the present systematic review [20,21,22,23]. The protocol of the present systematic review was registered on Open Science Framework [https://osf.io/xu8zq/ (accessed on 11 March 2025)].

### 2.2. Eligibility Criteria

For this systematic review, the research question was as follows: “does allergen sensitization affect orthodontic tooth movement-associated phenomena”? Based on the PICOS approach, specific selection criteria were used for the domains of study design, participant characteristics, intervention characteristics, primary outcome measures, and the types of study designs. Specifically, the following criteria were used:

1. Population (P): Every kind of experimental animal having undergone any kind of allergen sensitization.
2. Intervention (I): Any kind of orthodontic intervention.
3. Comparator (C): Animals without allergen sensitization but receiving orthodontic intervention.
4. Outcomes (O): Primarily, root resorption (extent/severity; assessed using linear measurements or grading scales or percentages); secondarily, the amount of tooth movement (assessed with filler gauges, CT, on histological sections), histologic changes, and biomolecular levels.
5. Study design (S): Prospective controlled studies (case-control, cohort etc.).

Human studies; ex vivo, in vitro, in silico, non-comparative studies (case reports and case series); and reviews (traditional reviews, systematic reviews, and meta-analyses) were excluded from further consideration. If tooth movement was performed in conjunction with additional interventions, such as tooth extraction, etc., on subjects after the cessation of active orthodontic tooth movement or subjects with other co-morbidities, then these studies were eliminated.

### 2.3. Information Sources and Search Strategy

For each database, EGK and FSB created detailed search strategies (Table 1). Their basis was the strategy developed for MEDLINE which was modified for differences in the controlled vocabulary and syntax rules of each database to be taken into consideration. MEDLINE via PubMed, CENTRAL, Cochrane Database of Systematic Reviews, Scopus, Web of Science Core Collection, and ProQuest Dissertations & Theses Global database were searched from inception to 23 December 2024.

There were no restrictions on the language, publication date, or status. Where possible, proceedings and abstracts from conferences were obtained, and the reference lists of all eligible studies were searched for more records.

### 2.4. Study Selection

FSB and EGK independently evaluated the retrieved records for inclusion, without being blinded to the authors’ identities, institutions, or research findings. The full texts of the records that were thought to meet the inclusion criteria by either reviewer were obtained and independently evaluated, while disagreements were resolved through discussion with another author (AEA).

### 2.5. Data Collection and Data Items

The authors FSB and MAM extracted data independently, and any disagreements were addressed through discussion with SK. Data collection forms included the following pieces of information: the bibliographic details of the study, details on the study design and verification of study eligibility, subject characteristics (where available, number, weight, age, etc.), sensitization methodology, tooth movement model, and details on outcome characteristics and results. If clarifications or additional materials were required regarding the published data, attempts were made to contact the corresponding authors.

### 2.6. Risk of Bias in Individual Studies

Using the SYRCLE’s risk of bias tool [24], FSB and MAM individually and in duplicate evaluated the risk of bias of the studies during the data extraction process. Disagreements were settled through discussion with another author (EGK).

After entering the information reported in each study into the data extraction form, each domain was assigned a risk of bias rating of low, high, or unclear [25].

### 2.7. Synthesis of Results, Risk of Bias Across Studies, and Additional Analyses

Even though a synthesis of the results was planned according to the research protocol, it was not carried out in the end due to a lack of sufficient data. Moreover, analyses for “small-study effects” and publication bias could not be performed [24], nor could the quality of evidence using the Grades of Recommendation, Assessment, Development, and Evaluation (GRADE) approach be assessed due to the insufficient number of trials that could be retrieved [26].

## 3. Results

### 3.1. Study Selection

**Figure 1:**
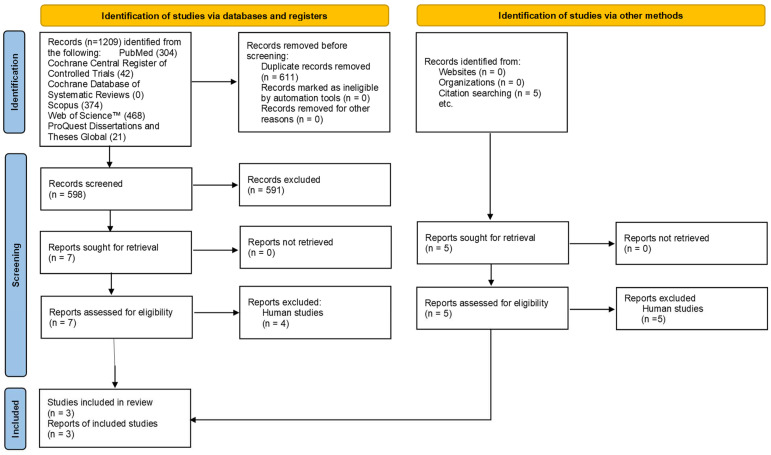
Flow chart of records.

A flowchart of records is shown in Figure 1. Initially, 1209 records were found from databases and another 5 from citation searching; all records from citation searching were excluded. A total of 611 of the records identified in databases were duplicates, and 595 were eliminated, 4 of which were human studies [6,27,28,29]. Finally, three full-text animal studies were included in this systematic review [30,31,32].

### 3.2. Study Characteristics

Table 2 summarizes the features of the included publications. All retrieved studies were of a cohort design and included allergen sensitization via ovalbumin. The duration and amount of orthodontic forces differed among the studies. Machado et al. (2012) used 40 g of force for three days, which started the day after the last ovalbumin administration [31]. Murata and co-workers (2013) started to apply 10 g of force, 7 days after the last sensitization, with the force being applied for 21 days [32]. Aghili et al. (2013) used 50 g of force, for 2 weeks, starting the day after ovalbumin sensitization. In this case, ovalbumin was also applied weekly during the period of force application [30].

Whereas Aghili et al. (2013) [30] studied the effects in Wistar rats, Machado et al. (2012) [31] investigated the initial stages of OTM by analyzing the histomorphologic characteristics of the PDL in Wistar rats.

Murata et al. (2013) focused primarily on the effects of allergen sensitization on OIRR and OTM in Brown Norway rats [32]. They used a digital microscope to measure the area of OIRR as well as the number of odontoclasts and osteoclasts within a specific area of the right first molar distopalatal root. The amount of OTM was measured in micro-CT images as the distance from the distal surface of the first molar to the mesial surface of the second molar. ELISA was used to measure the levels of RANKL, TNF-α, IL-1β, IL-6, and leukotrienes and related synthases and receptors in the periodontal tissues surrounding the first molar. Aghili and co-workers (2013) used optical microscopy to calculate root resorption as the ratio of the total length of resorptive lacunae to the length of the root circumference in sections of the right first molar mesial root [30]. Machado et al. (2012) measured the area of the PDL of the first molar mesial root using optical microscopy [31].

Finally, the study by Murata et al. (2013) [32] was supported by grants, offered by the Japanese Ministry of Education, Culture, Sports, Science and Technology, whereas the source of support for the research conducted by Aghili et al. [30] was the Vice chancellery of research, Shahid Sadoughi University of Medical Sciences, Yazd, Iran.

### 3.3. Risk of Bias Within Studies

Table 3 presents a summary of the risk of bias assessment. Most of the evaluated domains were determined to have a high risk of bias. The risk of bias was determined to be low in the domain of baseline similarity.

### 3.4. Results of Individual Studies

Allergen sensitization was successful in all included studies. Leukocyte and eosinophil counts increased in the samples retrieved from experimental animals [31,32].

Murata and co-workers (2013) found that root resorption was significantly greater in ovalbumin-sensitized animals (*p* < 0.05) [32]. Aghili et al. (2013) reported that although the percentage of total root resorption increased in sensitized animals (mean ± SD: 33.5 ± 5.7) compared to the control group (24.4 ± 8.5), the difference was not significant (*p* > 0.05) [30].

Regarding the amount of OTM, Murata et al. (2013) found that it was considerably greater in sensitized animals in comparison to the control group (*p* < 0.05) [32]. Similarly, Machado and co-workers (2012) observed that initial orthodontic movement was more pronounced in the experimental group [31].

The number of odontoclasts and osteoclasts was reported to increase in the ovalbumin-sensitized animals one week after the initiation of force application (*p* < 0.05). Odontoclast levels continued increase even 2 weeks later [32]. The levels of RANKL, TNF-α, and IL-1β were significantly higher in the ovalbumin-sensitized animals 24 h after the initiation of tooth movement (*p* < 0.05). IL-4 and IL-6 levels were also increased, although not reaching statistical significance [32]. The levels of leukotrienes, the synthases 5-LOX and LTA4 hydrolase, and the receptors BLT1 and BLT2 were significantly higher in the sensitized samples after 24 h of tooth movement [32].

## 4. Discussion

Asthma and airway allergies constitute entities of complex and multifactorial etiology, demonstrating the hypersensitivity of the tracheobronchial tree to a variety of stimuli. A combination of inherited and environmental factors plays a role in the creation of a mediated immune response, mostly via TH2 lymphocyte activation [33]. The investigation of bronchoalveolar lavage fluid from asthmatic patients reveals inflammatory reactions marked by a high number of mast cells, epithelial cells, eosinophils, and lymphocytes and a variety of inflammatory mediators [33].

The PDL demonstrates a comparable reaction during the initial stages of OTM [1]. Elucidating the implicated processes could possibly shed light on a potential inter-relationship between allergic sensitization and the phenomena associated with OTM and especially OIRR. The information retrieved showed the effects of allergen sensitization on the initial and subsequent OTM, while the results relevant to OIRR were contradictory. Even though several concerns were raised during the risk of bias assessment, necessitating the practitioner’s cautious approach to the findings, good practice suggests that orthodontists need to identify asthmatic or allergic patients and consider the potential implications.

In Wistar rats sensitized with ovalbumin, a histomorphometric investigation of the PDL during the early stages of orthodontic movement revealed improved responsiveness to mechanical stimuli. The PDL was more compressed in the pressure area and more stretched in the traction area, a reaction that could potentially be evidence of accelerated bone turnover and osteoclastogenesis/odontoclastogenesis [32].

Murata et al. (2013) assessed the rate of OTM and the amount of OIRR in ovalbumin-sensitized rats in comparison to control animals subjected to orthodontic force alone [32]. They discovered that the amount of movement and resorption was greater in the former group. Furthermore, RANKL and proinflammatory cytokines were shown to be overexpressed in the periodontal tissues of hypersensitive rats. Simultaneously, an increase in leukotriene B4 (LTB4), a potent lipid mediator of allergic inflammation, and the enzymes of the 5-lipoxygenase pathway, the leukotriene biosynthesis pathway, was detected. Additionally, they observed that low dosages of aspirin inhibited root resorption in allergen-sensitized rats, as well as RANKL, proinflammatory cytokines, and LTB4 expression. Contrary to the aforementioned results, Aghili et al. (2013) reported no statistically significant difference in root resorption development between sensitized and non-sensitized rats, although the percentage of resorption was increased in the former [30].

To calculate root resorption, numerous techniques have been used in several investigations. Some investigations, like Murata et al.’s (2013) [32], examined the root surface using longitudinal sagittal sections. Despite this technique detecting abnormalities from the cervical to apical areas, it cannot identify resorptions in the buccal and lingual regions. Aghili et al. (2013) analyzed buccolingual slices in the cervical and apical areas; however, defects may be underestimated because the section only passes through a restricted region [30]. To address this concern, at each level, three parallel parts with 150 μ intervals were passed. Others have used a grid to count the lines passing through the resorption area to determine its extent [34]. However, due to the ovoid geometry of the root, the buccal and lingual areas correspond to fewer gridlines, potentially underestimating OIRR in this area. The studies included in this systematic review used digital means to determine the defects’ boundaries and lengths, resulting in more objective measurements. Nevertheless, the diversity in the methodology used to assess resorption may have influenced the results reported. Differences in the types of histological sections, the locations of the sections, and the measurement procedures were identified.

Evidence regarding the associations of a medical history of asthma or allergy with the prevalence of OIRR in humans has also been found to be inconclusive [19]. McNab et al. (1999) [6], Nishioka et al. (2006) [35], and Malan (2017) [28] investigated the relationship between asthma and the development of root resorption following an orthodontic treatment with fixed appliances. Only McNab et al. (1999) identified a link between asthma medical history (both under medication and unmedicated) and a higher degree of OIRR of posterior teeth after controlling for potential variables [6]. Malan’s (2017) cohort analysis [28] and Nishioka and co-workers’ (2006) case–control research [35] found no evidence of an association.

Regarding the relationship between airway allergy and root resorption, Nishioka et al. (2006) observed that the incidence of allergy was significantly higher in the group with excessive root resorption [35]. Nanekrungsan et al. produced similar findings from a cohort design study [36]. Pastro et al. (2018), on the other hand, demonstrated no link between a history of allergies and moderate or excessive resorption in a case–control study design [29]. Owman-Moll and Kurol (2000) used a case–control approach to evaluate the role of allergies as a risk factor for root resorption development; individuals with allergies had a higher risk of root resorption, although the difference was not statistically significant [37].

Even though information from the few animal studies identified cannot be fully translated to human clinical scenarios, good practice would suggest that it is important to identify patients with asthma or airway allergy and consider the possible implications until more scientific information becomes available. In these circumstances, lower forces, more frequent consultations, and radiographic follow-up may be necessary, in addition to paying attention to the other factors related to root resorption development [38]. Mechanical or treatment-related risk factors for OIRR include tooth movement into the labial or cortical bone [39], long treatment duration and an increased magnitude of force [40,41], the amount of apical displacement [42], and the inclusion of extractions in the treatment plan [43]. Furthermore, biological or patient-related aspects should be taken into account. Some studies have found that root resorption associated with orthodontic treatment is more common in older patients [36,44]. Dental anomalies (ectopia, agenesis, taurodontism) [45,46]; teeth with pipette-shaped, blunt, abrupt deflection or narrow roots [41,46]; and parafunctional oral habits [43] should also receive appropriate attention.

### 4.1. Strengths and Limitations

Among the strengths of this systematic review is the adoption of a well-established methodology (based on the PRISMA statement). The technique used for data retrieval from electronic and manual sources was complete and comprehensive, with no pre-set constraints in terms of language, date, or publication status. In fact, the authors managed to retrieve all papers that could be potentially included in the present systematic review. In order to avoid potential biases, screening, eligibility verification, information abstraction, and bias risk assessment were all conducted in duplicate, and any disagreements were resolved through negotiation until a final agreement was reached. Finally, as relevant research on human subjects has considerable practical restrictions, the use of animal models may be advantageous [47,48].

The current review also has some limitations, which stem mostly from the nature and characteristics of the included studies as well as the data gathered throughout the review process. It should be noted that the data gathered are from a limited number of animal studies and therefore cannot be directly extrapolated to humans. There are significant variations between rats and humans, not just in bone physiology but also in response to allergens [49,50]. The sources of the risk of bias, including reporting bias, were detected in the included papers. Additional constraints affecting the precision of the retrieved results included the absence of relevant power sample calculations. Furthermore, the heterogeneity of these studies in conjunction with the utilization of specialized modalities to produce orthodontic tooth movement limits the generalizability of the information gained to human clinical scenarios.

### 4.2. Recommendations for Future Research

As extensive OIRR might have significant consequences and given the increased prevalence of asthma and airway allergies, more research is needed to investigate the phenomena associated with OTM thoroughly. It is also desirable that animal or even human study designs become standardized and that confounding factors and other potential sources of bias are addressed appropriately. Furthermore, future research should mimic, as closely as possible, scenarios in daily clinical practice applied to humans in terms of force magnitude as well as the properties of the force delivery system used. Also, future animal experiments could benefit from biomarker analyses, which would later be validated by clinical studies which would ideally follow a longitudinal cohort study design.

## 5. Conclusions

Despite indirect extrapolation to humans, the evidence from the limited number of retrieved animal studies suggests a potential impact of allergen sensitization on the phenomena associated with orthodontic tooth movement. Until further data become available, good practice would suggest identifying people with asthma or allergies and evaluating the potential implications.

## Figures and Tables

**Table 1:** Strategy for database search.

Database [23 December 2024]	Search Strategy	Hits
PubMed	(orthodon* OR “orthodontic force” OR “mechanical force”) AND (“tooth movement” OR “orthodontic movement” OR “orthodontic anchorage” OR “root resorption”) AND (allergy OR allergic* OR sensitiv* OR hypersensitiv* OR ovalbumin OR OVA OR “Dust Mite” OR HDM OR ascaris OR aspergill* OR “cotton dust” OR latex OR DRA OR cockroach OR asthma* OR airway)	304
Cochrane Central Register of Controlled Trials	(orthodon* OR “orthodontic force” OR “mechanical force”) AND (“tooth movement” OR “orthodontic movement” OR “orthodontic anchorage” OR “root resorption”) AND (allergy OR allergic* OR sensitiv* OR hypersensitiv* OR ovalbumin OR OVA OR “Dust Mite” OR HDM OR ascaris OR aspergill* OR “cotton dust” OR latex OR DRA OR cockroach OR asthma* OR airway) in Title Abstract Keyword (word variations were searched)	42
Cochrane Database of Systematic Reviews	(orthodon* OR “orthodontic force” OR “mechanical force”) AND (“tooth movement” OR “orthodontic movement” OR “orthodontic anchorage” OR “root resorption”) AND (allergy OR allergic* OR sensitiv* OR hypersensitiv* OR ovalbumin OR OVA OR “Dust Mite” OR HDM OR ascaris OR aspergill* OR “cotton dust” OR latex OR DRA OR cockroach OR asthma* OR airway) in Title Abstract Keyword (word variations were searched)	0
Scopus	TITLE-ABS-KEY ((orthodon* OR “orthodontic force” OR “mechanical force”) AND (“tooth movement” OR “orthodontic movement” OR “orthodontic anchorage” OR “root resorption”) AND (allergy OR allergic* OR sensitiv* OR hypersensitiv* OR ovalbumin OR ova OR “Dust Mite” OR hdm OR ascaris OR aspergill* OR “cotton dust” OR latex OR dra OR cockroach OR asthma* OR airway))	374
Web of Science™	(orthodon* OR “orthodontic force” OR “mechanical force”) AND (“tooth movement” OR “orthodontic movement” OR “orthodontic anchorage” OR “root resorption”) AND (allergy OR allergic* OR sensitiv* OR hypersensitiv* OR ovalbumin OR OVA OR “Dust Mite” OR HDM OR ascaris OR aspergill* OR “cotton dust” OR latex OR DRA OR cockroach OR asthma* OR airway) (Topic) and Preprint Citation Index (Exclude—Database)	468
ProQuest Dissertations and Theses Global	title((orthodon* OR “orthodontic force” OR “mechanical force”) AND (“tooth movement” OR “orthodontic movement” OR “orthodontic anchorage” OR “root resorption”) AND (allergy OR allergic* OR sensitiv* OR hypersensitiv* OR ovalbumin OR OVA OR “Dust Mite” OR HDM OR ascaris OR aspergill* OR “cotton dust” OR latex OR DRA OR cockroach OR asthma* OR airway)) OR abstract((orthodon* OR “orthodontic force” OR “mechanical force”) AND (“tooth movement” OR “orthodontic movement” OR “orthodontic anchorage” OR “root resorption”) AND (allergy OR allergic* OR sensitiv* OR hypersensitiv* OR ovalbumin OR OVA OR “Dust Mite” OR HDM OR ascaris OR aspergill* OR “cotton dust” OR latex OR DRA OR cockroach OR asthma* OR airway)) [Full text]	21

**Table 2:** The characteristics of the included studies.

Study	Animal Characteristics and Allergen Sensitization Method	Group Characteristics	Tooth Movement Model	Measurement Methodology
Machado et al. 2012 [31]	**Wistar rats** male, 180–200 g **OVA + Al(OH)_3_:** SC; on d 1 and 14 [reinforcement] **OVA:** NI; for 3 d [7 d after reinforcement]	EG1: 8; OVA EG2: 8; OVA + OTM CG1: 8 CG2: 8; OTM **Sample size calculation:** NM	NiTi CCS from L Mx I to FM [40 g] **Force application:** - Initiated 1 d after OVA sensitization. - Lasted for 3 d.	**Histological analysis:** OTM: PDL area [FM mesial root]
Murata et al. 2013 [32]	**Brown Norway rats** male, 6 w old, 110–140 g **OVA + Al(OH)_3_:** SC; on d 1 **OVA:** IP; 7 d later	EG1: 7; OVA EG2: 14; OVA + OTM EG3: 14; OVA + OTM + aspirin CG1: 7 CG2: 14; OTM **Sample size calculation:** NM	NiTi CCS from R Mx I to FM [≅10 g] **Force application:** - Initiated 7 d after OVA sensitization. - Lasted for 2 w.	**Histological analysis:** RR [area]; number of odontoclasts and osteoclasts [FM distopalatal root] **Micro-CT:** OTM [μm]: distal of FM to mesial of SM **ELISA:** TNF-α, IL-1β, IL-6, RANKL, leukotrienes, synthases 5-LOX and LTA4 hydrolase, receptors BLT1 and BLT2
Aghili et al. 2013 [30]	**Wistar rats** male, 3 m old, 330–350 g **OVA + alume:** IP; daily for 3 ds **OVA:** IP; weekly for 3 w	EG1: 15; OVA EG2: 15; OVA + OTM CG1: 15 CG2: 15; OTM **Sample size calculation:** NM	NiTi CCS from R Mx I to FM [50 g] Force application:* - Initiated 1 d after OVA sensitization. - Lasted for 2 w. * OVA was also applied weekly [IP] for 2 w during force application	**Histological analysis:** RR [%]; sum of length of resorptive lacunae to mean length of root periphery [FM mesial root]

BLT: branchless trichome; CCS: closed coil spring; CG: control group; d: day (s); EG: experimental group; FM: first molar; I: incisor; g: grams; IP: intraperitoneally; m: month; LOX: lysyl oxidase; LTA: lymphotoxin alpha; Mx: maxillary; NI: nasal instillation; NM: not mentioned; R: right; RANKL: Receptor activator of nuclear factor kappa-Β ligand; RR: root resorption; SC: subcutaneously; SM: second molar: OTM: orthodontic tooth movement; OVA: ovalbumin: PDL: periodontal ligament; TNF: tumor necrosis factor; w: week; %: percentage.

**Table 3:** Risk of bias assessment.

	Signaling Questions									
Study	1	2	3	4	5	6	7	8	9	10
Machado et al. 2012 [31]	high	low	high	high	high	high	high	low	unclear	unclear
Murata et al. 2013 [32]	high	low	high	high	high	high	high	low	unclear	unclear
Aghili et al. 2013 [30]	unclear	low	unclear	high	high	high	high	unclear	unclear	unclear

1: Was the allocation sequence adequately generated and applied? 2: Were the groups similar at baseline, or were they adjusted for confounders in the analysis? 3: Was the allocation adequately concealed? 4: Were the animals randomly housed during the experiment? 5: Were the caregivers and investigators blinded to the intervention that each animal received? 6: Were animals selected at random for outcome assessment? 7: Was the outcome assessor blinded? 8: Were incomplete outcome data adequately addressed? 9: Are the reports of the study free of selective outcome reporting? 10: Was the study apparently free of other problems that could result in a high risk of bias?
